# The prognostic significance of global aberrant alternative splicing in patients with myelodysplastic syndrome

**DOI:** 10.1038/s41408-018-0115-2

**Published:** 2018-08-13

**Authors:** Yi-Tsung Yang, Yu-Chiao Chiu, Chein-Jun Kao, Hsin-An Hou, Chien-Chin Lin, Cheng-Hong Tsai, Mei-Hsuan Tseng, Wen-Chien Chou, Hwei-Fang Tien

**Affiliations:** 1 Division of Hematology-Oncology, Department of Internal Medicine, National Taiwan University Hospital Hsin-Chu Branch, Hsinchu, Taiwan; 2 Graduate Institute of Clinical Medicine, College of Medicine, National Taiwan University, Taipei, Taiwan; 3 Greehey Children’s Cancer Research Institute, UT Health San Antonio, San Antonio, TX USA; 4 Division of Hematology, Department of Internal Medicine, National Taiwan University Hospital, Taipei, Taiwan; 5 Laboratory Medicine, National Taiwan University Hospital, Taipei, Taiwan; 6 Tai-Chang Stem Cell Therapy Center, National Taiwan University, Taipei, Taiwan

## Abstract

Aberrant alternative splicing (AS) is a hallmark of cancer development. However, there are limited data regarding its clinical implications in myelodysplastic syndrome (MDS). In this study, we performed an in-depth analysis of global AS in 176 primary MDS patients with 20 normal marrow transplant donors as reference. We found that 26.9% of the expressed genes genome-wide were aberrantly spliced in MDS patients compared with normal donors. These aberrant AS genes were related to pathways involved in cell proliferation, cell adhesion and protein degradation. A higher degree of global aberrant AS was associated with male gender and *U2AF1* mutation, and predicted shorter overall survival and time to leukemic change. Moreover, it was an independent unfavorable prognostic factor irrespective of age, revised international prognostic scoring system (IPSS-R) risk, and mutations in *SRSF2*, *ZRSR2*, *ASXL1*, *TP53*, and *EZH2*. With LASSO-Cox regression method, we constructed a simple prognosis prediction model composed of 13 aberrant AS genes, and demonstrated that it could well stratify MDS patients into distinct risk groups. To our knowledge, this is the first report demonstrating significant prognostic impacts of aberrant splicing on MDS patients. Further prospective studies in larger cohorts are needed to confirm our observations.

## Introduction

Alternative splicing (AS) is a physiological phenomenon to ensure higher protein diversity for better environmental fit. Around 70% of human genes undergo AS and 95% of multi-exonic genes are alternatively spliced^1^. Splicing is a complex and tightly regulated process. Perturbation of this process by spliceosome gene mutations, epigenetic modifications, or other causes would lead to aberrant AS, resulting in deregulation of many cellular processes, such as cell proliferation, adhesion, differentiation, motility, invasion, and death^2,3^.

Evidence has shown that aberrant AS is a hallmark of cancer development^4^. A recent study demonstrated that approximately 29% of the expressed genes genome-wide were aberrantly spliced in acute myeloid leukemia (AML) patients compared with normal donors^5^. The genes with aberrant AS encode several oncoproteins, tumor suppressor proteins, ribonucleoproteins, and proteins involved in apoptosis, cell proliferation, and spliceosome assembly^5,6^. These findings provide a link between aberrant AS and AML pathogenesis.

Myelodysplastic syndrome (MDS) is a highly heterogeneous disease whose pathogenesis is still under investigation. The pattern of global aberrant AS and its clinical implications in MDS are not well known yet. We wonder if aberrant AS would be related to the pathogenesis and clinical features of MDS. To answer these questions, we determined the global gene expression and splicing patterns from a large cohort of de novo MDS patients, using normal transplant donors as reference. With complete clinical and genetic data of these patients, we were able to analyze the clinical and pathological implications of global aberrant AS for MDS patients.

## Materials and methods

### Study design and patient cohort

From November 1991 to December 2010, a total of 176 primary MDS patients who were diagnosed at the National Taiwan University Hospital (NTUH) and had available samples and complete clinical and gene mutation data were enrolled in this study. Twenty healthy bone marrow (BM) donors of hematopoietic stem cell transplantation (HSCT) in this period were collected as normal controls. We also recruited another cohort of 31 primary MDS patients diagnosed between January 2011 and May 2012 as a validation cohort to confirm the prognostic impact of aberrant AS. The patients were reclassified according to the 2016 World Health Organization (WHO) classification^7^. All patients gave their informed consent to participate in the study. This study was approved by the Institutional Review Board of the NTUH.

### Cytogenetic and mutation analyses

Chromosome abnormalities^8^ and genetic mutation analyses were performed as previously reported^9–18^.

### Affymetrix HTA 2.0 analysis

The RNA was extracted from BM mononuclear cells (BMMNCs) by the TRIzol method at initial MDS diagnosis. RNA concentration and integrity were evaluated with ND-1000 spectrophotometer (NanoDrop Technologies, Wilmington, DE, USA) and 2100 Bioanalyzer (Agilent Technologies, Palo Alto, CA, USA). One microgram of RNA from each sample was processed with the miRNeasy mini kit (Qiagen, Hilden, Germany) and then it was labeled with biotin, followed by hybridization to Affymetrix GeneChip Human Transcriptome Array 2.0 (HTA 2.0) (Affymetrix, Santa Clara, CA), and finally scanned at Microarray Core Lab of the National Health Research Institutes according to Affymetrix protocols. We transformed the intensities to digital signals by Affymetrix AGCC software for further analyses with the Affymetrix Expression Console software and Transcriptome Analysis Console (TAC) version 3.0 software (flowchart in Supplementary Figure S1). The raw and normalized microarray data of Affymetrix HTA 2.0 reported in this article have been deposited in the Gene Expression Omnibus database (accession number GSE107400).

### Definition of the parameters for aberrant splicing

We used the splicing index (SI) method based on default setting of TAC version 3.0 software to define the aberrant AS events of MDS patients with normal control donors as reference^19,20^. A probe selection region (PSR) is a region with clustering probes in an exon. The SI of a PSR is derived from the ratio of the PSR signal intensity between MDS patients and normal control donors after normalization by the gene expression levels, i.e.,

$${\mathrm{Splicing}}\,{\mathrm{index}}\,{\mathrm{of}}\,{\mathrm{RSRa}} = \frac{{\left[ {\frac{{\mathrm{{Intensity}}\,{\mathrm{of}}\,{\mathrm{PSRa}}\,{\mathrm{in}}\,{\mathrm{gene}}\,{\mathrm{A}}}}{{{\mathrm{gene}}\,{\mathrm{A}}\,{\mathrm{expression}}\,{\mathrm{level}}}}} \right]{\mathrm{of}}\,{\mathrm{MDS}}\,{\mathrm{patient}}}}{{\left[ {\frac{{{\mathrm{Intensity}}\,{\mathrm{of}}\,{\mathrm{PSRa}}\,{\mathrm{in}}\,{\mathrm{gene}}\,{\mathrm{A}}}}{{{\mathrm{gene}}\,{\mathrm{A}}\,{\mathrm{expression}}\,{\mathrm{level}}}}} \right]{\mathrm{of}}\,{\mathrm{normal}}\,{\mathrm{donor}}}}$$

When the value is less than 1, SI is defined as the negative reciprocal. For example, an SI value of 1/4 is converted to −4. SI has to be < −2 or > 2 to be denoted as an aberrant AS event. Each gene may have more than one aberrant AS event, depending on how many PSRs with abnormal SI in that gene. A gene with at least one aberrant AS event is called an aberrant AS gene.

Next, we defined aberrant AS score as genome-wide total aberrant AS events divided by total aberrant AS genes in each MDS patient. Therefore, a higher aberrant AS score indicated more aberrant AS events in each aberrant AS gene on average, which implied a higher degree of aberrant AS.

### Bioinformatics analysis

Functional annotation for the aberrant AS genes of whole genome was performed by the Database for Annotation, Visualization and Integrated Discovery (DAVID) web tool with default settings to comprehensively explore the dysregulated functional relevance of these genes.

We sought to construct a concise prognosis prediction model out of the aberrant AS events. All analyzable SI values of PSRs were log2 transformed and analyzed for the association with overall survival (OS) by the univariate Cox proportional hazards model. The prognostic aberrant AS events identified were then merged into a Least Absolute Shrinkage and Selection Operator (LASSO) analysis in the Cox model (LASSO-Cox)^21^. LASSO-Cox is a regression method that selects a subset of variables (i.e., aberrant AS events) to yield the best prediction of OS while reducing overfitting of data. Here the optimal subset of aberrant AS events was determined by a leave-one-out cross validation analysis within the training cohort. As a result, LASSO-Cox assigned a regression coefficient to each of the selected aberrant AS events. Based on the results, we constructed a scoring system by a weighted sum of the selected events, where the weights were LASSO regression coefficients. The scoring system was applied to predict OS and time to leukemic change (TTLC) (flowchart in Supplementary Figure S2). With further analysis using the Geneious version 10.2.2 software (Biomatters, New Zealand), we could predict the consequence of protein changes by aberrant AS events among these aberrant AS genes.

### Validation of the array data

Direct PCR followed by TA cloning with Taq polymerase-amplified (TA)-cloning vector pGEM®-T Easy (Promega, Madison, WI, USA) and sequencing or real time (RT)-PCR were performed to validate aberrant AS events detected by Affymetrix HTA 2.0. We used cDNA samples generated from total RNA extracted from BMMNCs of randomly selected MDS patients for validation. We chose the genes with high SI values and similar expression levels between the MDS patients and normal control donors for validation. *AUP1* gene fulfilling the criteria was randomly chosen for further validation. Besides, another 2 genes *PVRL2* and *GRIK5* in LASSO-Cox regression model were also randomly picked for validation. *GAPDH* was selected as the internal control gene.

All the aberrant splicing transcripts were verified using cDNA of BMMNCs in 4 MDS patients and 3 normal control donors. *AUP1* transcript was validated through PCR followed by TA cloning and sequencing. *PVRL2* and *GRIK5* were validated by RT-PCR method. The primer pairs used were shown below:

*AUP1:* forward (5′-AAGGAAGTTTTGCCCCATGT-3′) at exon 9

reverse (5′-CCCCCTCAAGCAGATTAGTG-3′) at exon 10

*PVRL2:* forward (5′-CCCTCCTGAAGTGTCCATCT-3′) at exon 3

reverse (5′-TGCTCCAGTCATAGCCCGTG-3′) at exon 4

*GRIK5:* forward (5′-TTCGTGGCGGTCATGGAATT-3′) at exon 18

reverse (5′-TTGCTGAGGCGCATCTCG-3′) at exon 19

Due to different gene expression levels of *GRIK5* between MDS patients and normal donors (fold change 2.9) at array raw data, verification of overall *GRIK5* gene expression was performed using another primer pair (5′-CCGGGATGAGATCACACTGG-3′) and (5′-TGACCTCCGTGTGGACCATA-3′) covering exon 17 and exon 18.

### Statistical analysis

All statistical analyses were performed by SPSS version 17.0 software (SPSS Inc., Chicago, IL, USA). The ANOVA test was used for comparison of aberrant AS scores among different MDS subgroups based on French-American-British (FAB) or World Health Organization (WHO) classification or genetic mutations. The Pearson correlation analysis was applied for the test of correlation between global aberrant AS scores with clinical and biological data. The Kaplan-Meier method was used to evaluate median OS and TTLC. OS was estimated from the date of first MDS diagnosis to the date of last follow-up or death due to any cause, and TTLC was measured as the duration from the date of initial MDS diagnosis to the date of acute leukemic change. Log-rank test was used to compare median OS and TTLC among different groups according to global aberrant AS scores and LASSO-Cox regression model. Univariate and multivariate Cox proportional hazard regression analysis were used to investigate independent prognostic factors for OS and TTLC. A *P*-value < 0.05 was considered statistically significant.

## Results

### Clinical characteristics of the patients

Among the 176 MDS patients recruited in this study, most (84.1%) received only supportive care. Four patients (2.3%), two patients (1.1%), and 22 patients (12.5%) received intensive chemotherapy, hypomethylating agents, and allogeneic HSCT, respectively. The baseline characteristics of these patients were summarized in Table 1.

**Table 1:** Baseline characteristics of 176 patients with primary MDS

Characteristics	Total number
*Age (ranged from 19 to 94 years, median 69 years)*	
≧65 years	102 (58%)
<65 years	74 (42%)
*Gender*	
Male	121 (69%)
Female	55 (31%)
*FAB*	
RA	78 (44%)
RARS	22 (13%)
RAEB	76 (43%)
*WHO 2016*	
MDS-SLD	40 (23%)
MDS-MLD	38 (22%)
MDS-RS-SLD	13 (7%)
MDS-RS-MLD	9 (5%)
MDS-EB1	32 (18%)
MDS-EB2	44 (25%)
*IPSS-R (only 164 patients could be evaluated)*	
Very low	6 (4%)
Low	54 (33%)
Intermediate	41 (25%)
High	35 (21%)
Very high	28 (17%)

*FAB* French-American-British classification, *WHO* World Health Organization, *IPSS-R* revised international prognostic scoring system, *MDS* myelodysplastic syndrome, *SLD* single lineage dysplasia, *MLD* multilineage dysplasia, *RS* ring sideroblasts, *EB* excess blasts

### Aberrant splicing of whole transcriptome

This Affymetrix HTA 2.0 contains over 6 million distinct probes covering transcripts of 44,710 coding genes, 22,829 non-coding genes, and spanning the exon-exon junctions (ten probes per exon; four probes per exon-exon junction). As a whole, there are 670,402 PSRs and 339,146 splice junction probe sets among 378,502 exons in 67,539 genes for evaluation by this array platform. This comprehensive coverage of the exons and junctions enables us to simultaneously determine gene expression levels and splicing patterns of every gene.

Totally 52,646 of 67,539 (77.9%) genes on the array were expressed in BMMNCs from both MDS patients and normal controls. Approximately 26.9% (14,162 of the 52,646) of the expressed genes were aberrantly spliced in the MDS patients, that is, these genes showed at least one aberrant AS event, compared with normal donors. Besides, a total of 42,760 aberrant AS events among these 14,162 aberrantly spliced genes were noted in the whole transcriptome. In addition, 85.9% of these aberrant splicing events were mapped to coding genome and 14.1% to non-coding regions.

### Associations of clinical features with aberrant splicing

**Fig. 1:**
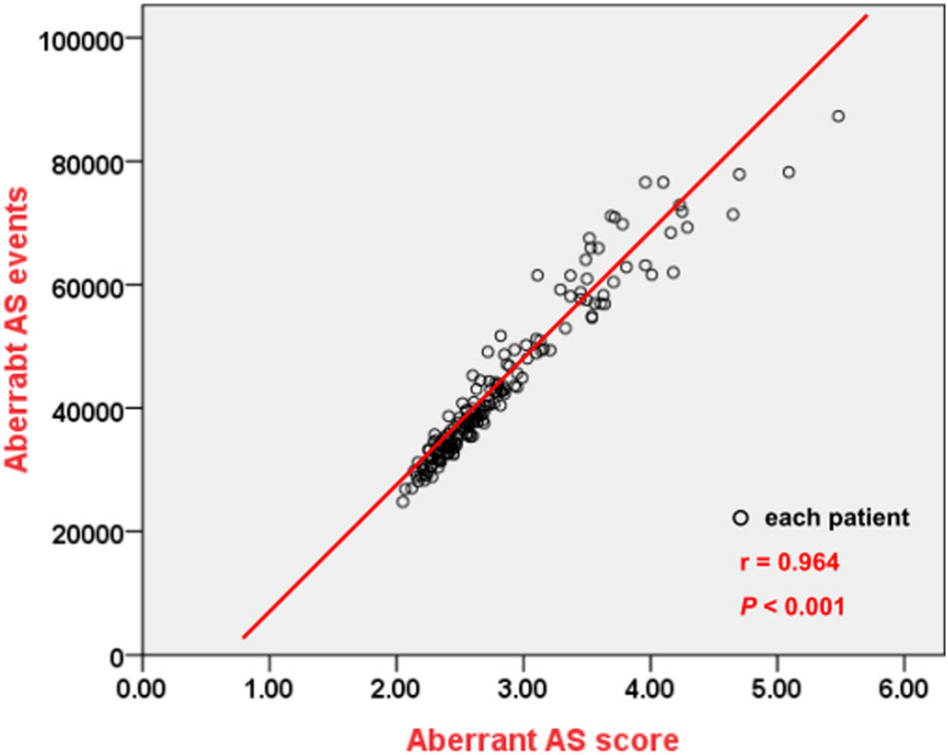
Aberrant AS scores and total aberrant AS events among MDS patients. Aberrant AS scores were strongly correlated with total aberrant AS events of whole transcriptome among 176 MDS patients

**Fig. 2:**
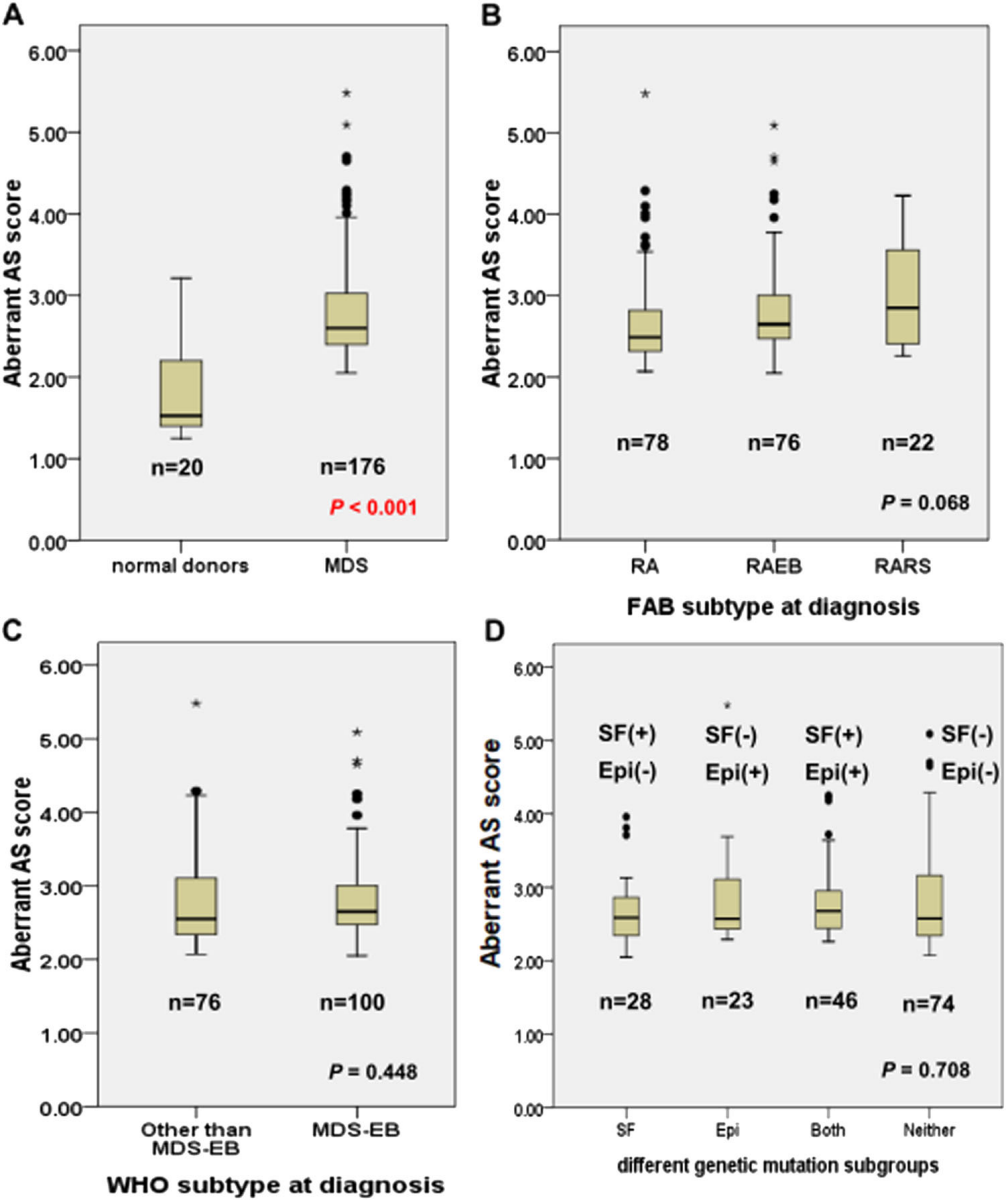
Aberrant AS scores among MDS patients. Aberrant AS scores in total cohort of 176 MDS patients and 20 normal donors (**a**), different MDS subgroups according to the FAB classification (**b**), 2016 WHO classification (**c**), and mutation status in splicing factor (SF) genes and epigenetic (Epi) genes (**d**). SF genes include four genes: *SF3B1*, *U2AF1*, *SRSF2*, and *ZRSR2*. Epigenetic genes include six genes: *TET2*, *ASXL1*, *DNMT3A*, *EZH2*, *IDH1* and *IDH2*. SF(+): at least one splicing factor gene mutation; Epi(+): at least one epigenetic gene mutation; SF(-): no splicing factor gene mutation; Epi(-): no epigenetic gene mutation; • Dots: mild outlier; * Stars: extreme outlier; bold horizontal lines: median

We calculated the aberrant AS score (i.e. total aberrant AS events divided by total aberrant AS genes) of each MDS patient with the 20 normal donors as reference. Our data displayed that aberrant AS scores were strongly correlated with total aberrant AS events among MDS patients (*r* = 0.964, *P* < 0.001, Fig. 1). Therefore, a higher aberrant AS score not only indicated more aberrant AS events in each aberrant AS gene on average, but also reflected more aberrant AS events in the whole transcriptome and would imply a higher degree of aberrancy of AS. We calculated the aberrant AS score of each normal control donor with the other 19 donors as reference. The score among the 20 normal donors ranged from 1.25 to 3.21 with a median of 1.525 (Fig. 2a). In contrast, the aberrant AS scores ranged from 2.05 to 5.48 with a median of 2.60 in MDS patients (Fig. 2a) indicating significantly higher aberrant AS scores among MDS patients than normal donors (*P* < 0.001). We used receiver operating characteristic (ROC) curve for survival prediction of MDS patients to obtain a cut-off point value at 2.45, which best differentiated the patients’ survival. We defined a low and high global aberrant AS score by this value.

A higher global aberrant AS score was significantly associated with male gender and *U2AF1* mutation (Table 2). But the aberrant AS scores were not significantly different among different MDS subgroups according to the FAB or 2016 WHO classification (Fig. 2b, c). They were also not significantly different among the patients classified by the status of mutations in genes related to spliceosome and epigenetic modifications (Fig. 2d, Supplementary Figure S3). There was no association between aberrant AS scores and age, hemogram, or cytogenetics, either (Table 2).

**Table 2:** Comparison of clinical manifestations between MDS patients with high and low global aberrant alternative splicing (AS) score

Variables	Total	High aberrant AS score	Low aberrant AS score	*P* value
	(*n*)	(*n*)	(*n*)	
*Sex* ^a^				0.024*
Male	121	87	34	
Female	55	30	25	
*Age (year)* ^b^	69 (19–94)	66 (19–89)	73 (26–94)	0.121
≧65	102	63	39	
<65	74	54	20	
*Laboratory data* ^b^				
WBC (/μL)	3825 (490–20440)	3780 (490–20440)	4660 (1710–11690)	0.426
Hb (g/dL)	8.1 (3.5–14.6)	8.1 (3.5–13.6)	8.1 (3.7–14.6)	0.792
Platelet (×1000 /μL)	85 (3–721)	82 (9–721)	106 (3–460)	0.08
*Cytogenetics* ^a^				
Favorable^c^	115	71	44	0.09
Intermediate^d^	23	17	6	0.382
Poor^e^	26	20	6	0.197
*Genetic alterations* ^a^				
*SF3B1*	29	17	12	0.342
*U2AF1*	14	13	1	0.028*
*SRSF2*	24	15	9	0.675
*ZRSR2*	15	12	3	0.254
*TET2*	22	17	5	0.246
*ASXL1*	36	26	10	0.386
*DNMT3A*	25	18	7	0.517
*EZH2*	10	7	3	0.809
*IDH1/IDH2*	4	4	0	0.151
*TP53*	12	10	2	0.193
*RUNX1*	25	18	7	0.517

*Statistically significant (*P* < 0.05)

^a^Number of patients

^b^Median (range)

^c^Favorable cytogenetics: -Y, del(11q), Normal, del(5q), del(12p), del(20q), double including del(5q)

^d^Intermediate-risk cytogenetics: del(7q), +8, +19, i(17q), any other single or double independent clones

^e^Poor-risk cytogenetics: −7, inv(3)/t(3q)/del(3q), double including −7/de(7q), complex: 3 abnormalities, complex: >3 abnormalities

### The prognostic impact of aberrant AS scores

**Fig. 3:**
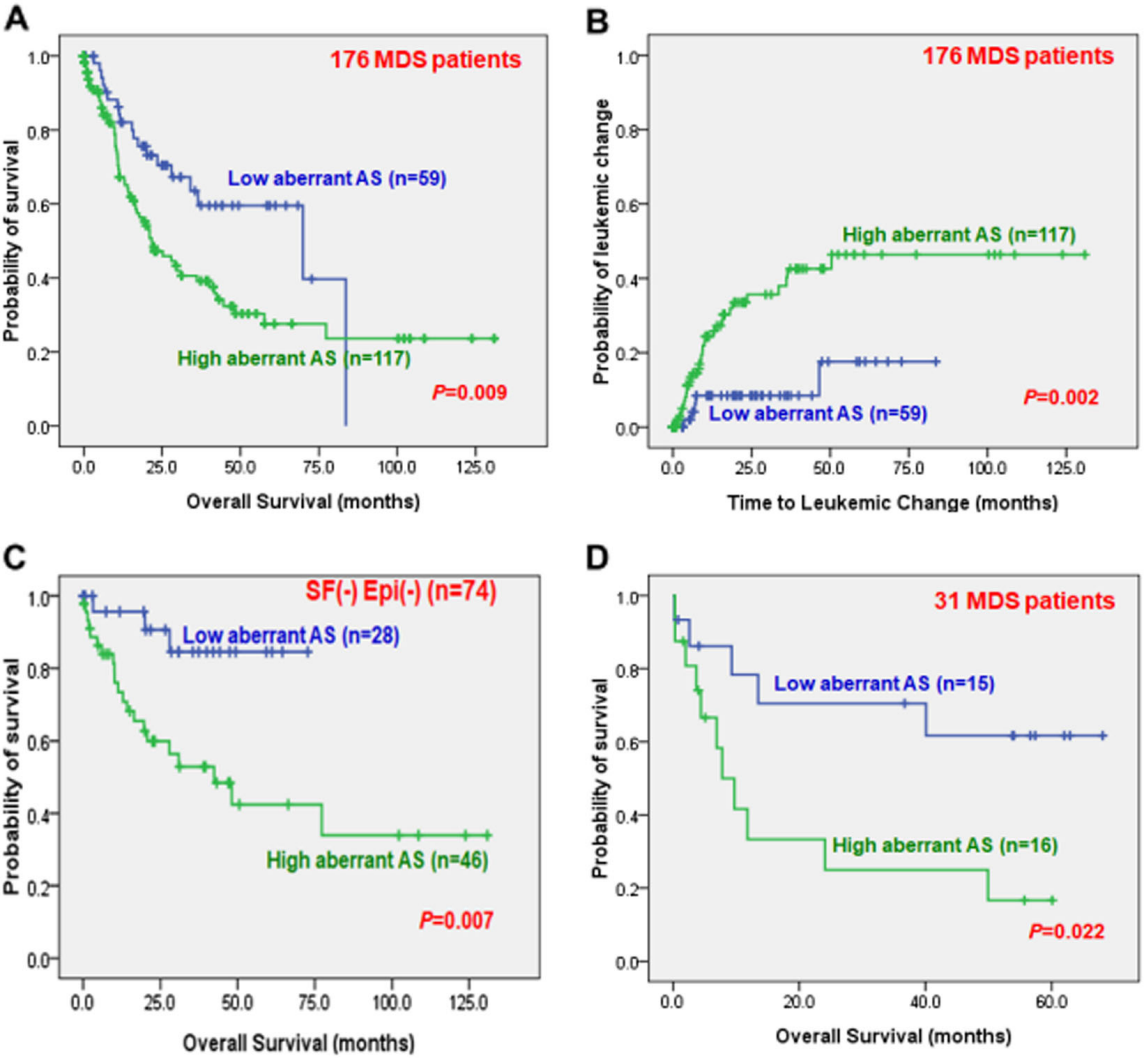
Prognostic impact of genome-wide aberrant AS pattern among MDS patients. Kaplan–Meier survival curves stratified by genome-wide aberrant AS pattern (with a cut-off point value at 2.45 of aberrant AS score) for overall survival (OS) (**a**) and time to leukemic change in total cohort of 176 MDS patients (**b**), OS in patients without any detectable mutation in genes related to splicing factors or epigenetic modifications (**c**), and OS in the validation cohort of 31 primary MDS patients (**d**). Note: SF(-)Epi(-) means neither splicing factor, nor epigenetic genes mutation

With a median follow up time of 48.4 months, a higher global aberrant AS score, indicating a higher degree of genome-wide aberrant AS pattern, predicted significantly shorter overall survival (OS) (median, 21.7 vs. 69.9 months, *P* *=* 0.009) and shorter time to leukemic change (TTLC) (first quartile, 12.5 months vs. not reached, *P* *=* 0.002) than those with a lower score (Fig. 3a, b). In order to clarify the pathological effects of aberrant splicing in the absence of mutations in either spliceosome complex or epigenetic modifiers, which were common in MDS patients^22^, we focused on subgroups of MDS patients without detectable mutations in genes related to spliceosome or epigenetic modifications and found that a higher global aberrant AS score could also predict shorter OS in these patients (Fig. 3c). Moreover, we were able to confirm that a higher global aberrant AS score could predict shorter OS in the validation cohort of 31 primary MDS patients (Fig. 3d).

Multivariate analysis including variables that had prognostic significance in univariate analysis (Supplementary Table S1) in the 176 MDS patients showed that a high global aberrant AS score was an independent unfavorable prognostic factor for both OS and TTLC irrespective of age, revised international prognostic scoring system (IPSS-R) risk, and mutations in *SRSF2*, *ZRSR2*, *ASXL1*, *TP53*, and *EZH2* (Table 3).

**Table 3:** Multivariate analysis (Cox regression) on the overall survival and time to leukemic change

Variables	Overall survival				Time to leukemic change			
	HR	95% CI		*P*	HR	95% CI		*P*
		Lower	Upper			Lower	Upper	
Age^a^	1.019	1.003	1.035	0.017*	0.984	0.965	1.005	0.133
IPSS-R^b^	3.114	1.839	5.270	<0.001*	3.694	1.710	7.982	0.001*
*SRSF2*	1.092	0.501	2.379	0.825	0.913	0.294	2.837	0.875
*ZRSR2*	1.400	0.665	2.950	0.376	0.690	0.212	2.242	0.537
*ASXL1*	1.628	0.784	3.381	0.191	4.788	1.933	11.857	0.001*
*EZH2*	1.227	0.456	3.300	0.685	0.701	0.171	2.881	0.622
*TP53*	4.563	1.936	10.753	0.001*	5.009	1.250	20.079	0.023*
Aberrant AS score^c^	1.801	1.019	3.181	0.043*	2.736	1.036	7.222	0.042*

*HR* hazard ratio, *CI* confidence interval

*Statistically significant (*P* < 0.05)

^a^Age as a continuous variable

^b^IPSS-R risk score > 4.5 relative to IPSS-R ≤ 4.5 (the reference)

^c^High global aberrant AS score relative to low global aberrant AS score (the reference)

Tefferi et al. recently developed a new prognostic model for primary MDS patients by integration of genetic and clinical information^23^. This Mayo Alliance Prognostic Model provides another platform for prognostication in MDS patients other than the IPSS-R. We were interested if the prognostic significance of aberrant splicing might hold up in the context of this new scoring system. We found that a higher degree of global aberrant AS was still an independent poor risk factor after adjustment for Mayo Alliance model by Cox regression analysis (Supplementary Table S2).

### Physiological significance of the aberrant splicing

To explore the physiological pathways perturbed by the aberrant splicing, we analyzed the genes with aberrant AS by DAVID database. We identified 71 signaling pathways significantly affected by aberrant splicing with *P* value < 0.001 and false discovery rate (FDR) < 0.05. We listed the top 20 pathways according to fold enrichment and discovered that pathways related to cell proliferation, adhesion, and protein degradation were affected by the aberrant AS (Supplementary Table S3). These findings indicated that the normal splicing mechanism was disrupted in MDS patients and the aberrant splicing in signaling networks seemed to be widespread which perturbed many normal physiological pathways.

### Aberrant AS events with prognostic significance

**Fig. 4:**
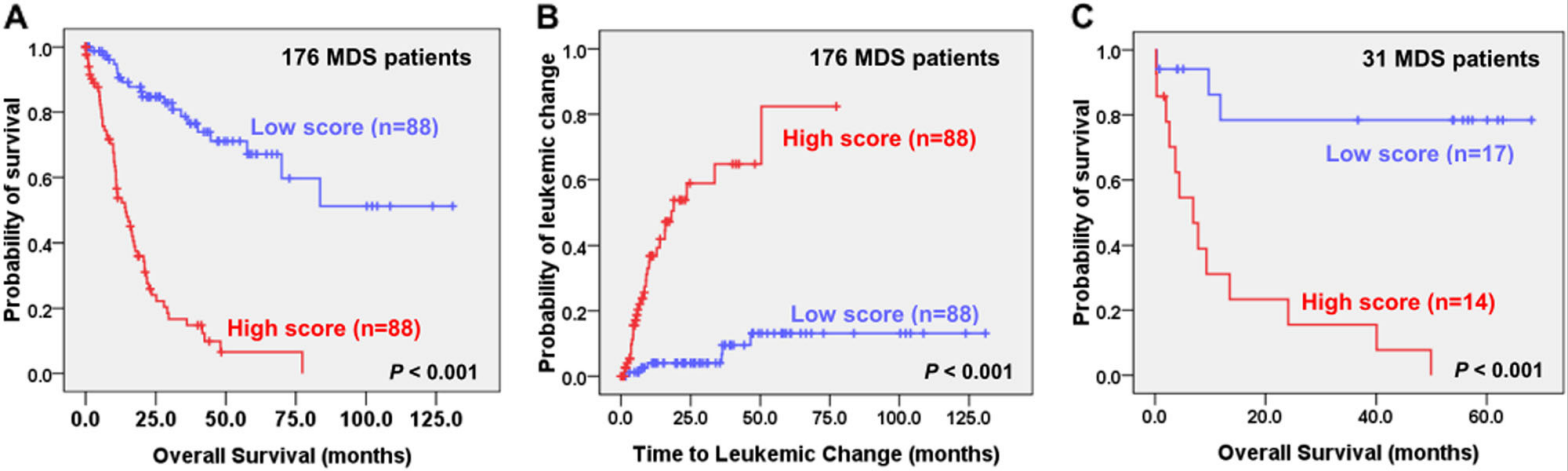
Prognostic impact of Lasso-Cox regression model among MDS patients. Kaplan–Meier survival curves stratified by the scoring system of LASSO–Cox regression model composed of 13 aberrant AS events for OS (**a**) and time to leukemic change (**b**) in total cohort of 176 primary MDS patients, and OS in validation cohort of 31 primary MDS patients (**c**)

We then sought to identify the core subsets of aberrant AS events that could predict the prognosis of MDS patients. A univariate Cox analysis identified 53 OS-associated aberrant AS events (Bonferroni adjusted *P* < 0.05). Among them, a subset of 13 aberrant AS events could best predict OS by a LASSO-Cox regression analysis. As a result, we developed a simple scoring system composed of a weighted sum of the SI of the 13 aberrant AS events on *ARHGEF17*, *C1QTNF4*, *EGFL7*, *GNAI1*, *GRIK5*, *HOXA9*, *KRT18*, *MAP3K15*, *MEG3*, *PTK7*, *PVRL2*, *PXDN*, and *TGFBI* (Supplementary Table S4). The scores of LASSO-Cox regression model ranged from −0.5 to 3.53 in our discovery cohort. With a cut-off point at the median value 1.14, a high score was significantly predictive of inferior OS (Fig. 4a; *P* < 0.001) and shorter TTLC (Fig. 4b; *P* < 0.001). The result was confirmed in the validation cohort (Fig. 4c; *P* < 0.001).

### Validation of the array-predicted splicing

**Fig. 5:**
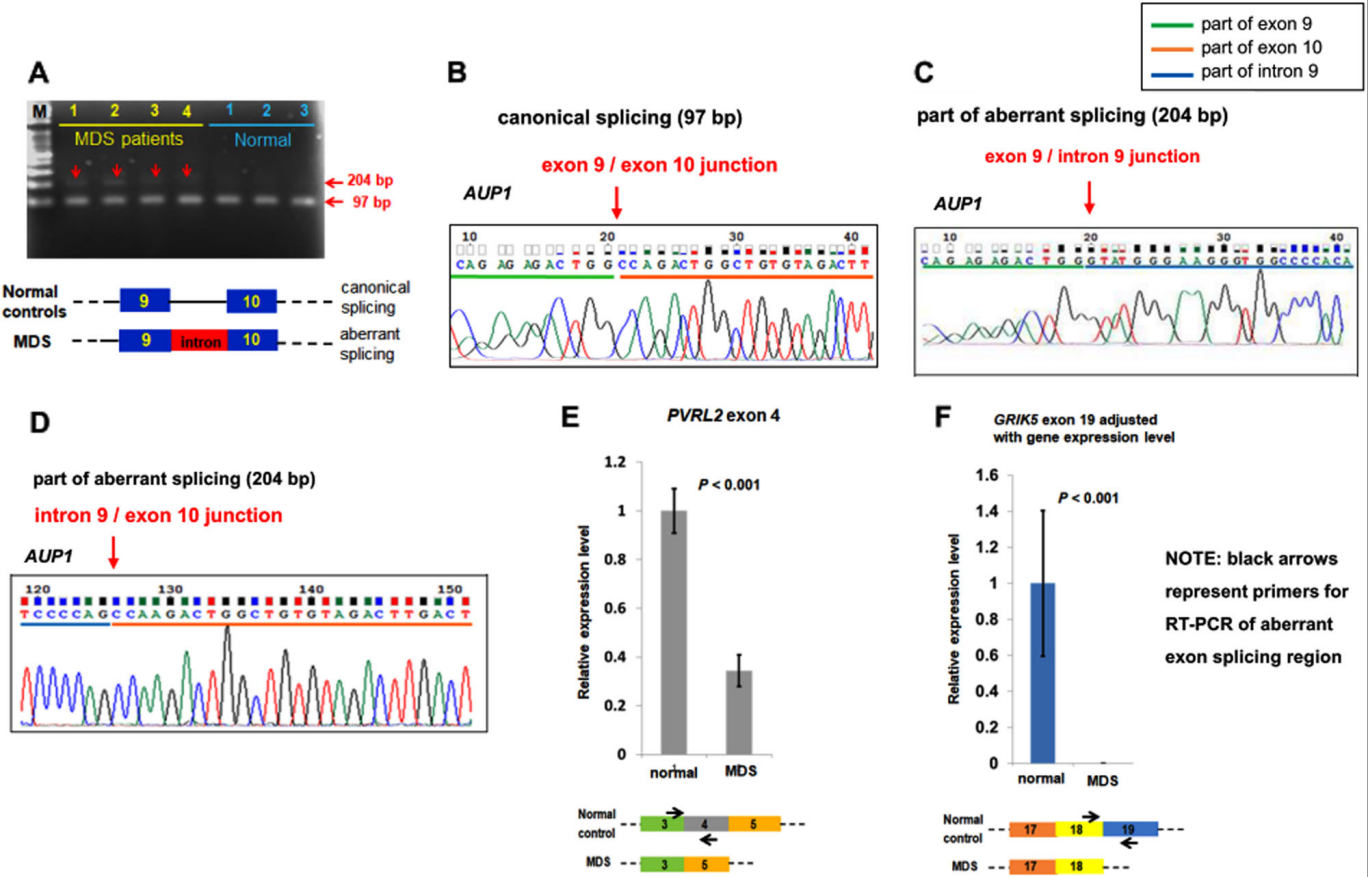
Validation studies of aberrant splicing in *AUP1*, *PVRL2*, and *GRIK5* specific transcripts. Validation of aberrant alternative splicing (AS) of *AUP1* transcript (NM_181575) in 4 MDS patients was performed by PCR of cDNA. An obvious additional band around 204 bp of *AUP1* specific-gene transcript was noted at samples from MDS patients compared with normal control donors (**a**). TA cloning and direct sequencing of the amplified cDNA collected from the canonical splicing 97 bp band (**b**) and the additional aberrant splicing 204 bp band from the MDS patient (**c** and **d**) successfully verified the aberrant intron retention between exon 9 and 10 in this gene. RT-PCR of *PVRL2* (**e**) and *GRIK5* (**f**) verified the aberrantly spliced transcripts (NM_001042724) and (NM_002088) respectively noted at HTA 2.0

To verify the aberrant AS events identified by the array, we chose 3 gene transcripts for validation as mentioned in Materials and Methods. *AUP1* gene is located at 2P13.1 and contains 12 exons; the transcript (NM_181575) showed aberrant retention of intron 9 (mean SI level 3.54) among most MDS patients (71.6%) in our cohort at HTA 2.0. An obvious additional band of *AUP1* specific transcripts at 204 base pairs was present in the 4 MDS patients studied, compared with 3 normal control donors (Fig. 5a). We verified the retention of intron 9 in *AUP1* in these MDS patients by direct sequencing (Fig. 5b–d). The aberrant AS of *AUP1* in MDS patients would lead to protein truncation by the prediction of Geneious software (Supplementary Figure S4). *PVRL2* gene (NM_001042724) is located at 19q13.32 and contains 9 exons; the array data showed exon 4 skipping in almost all of our MDS patients (98.3%). *GRIK5* gene (NM_002088) is located at 19q13.2 and contains 19 exons; the array data showed exon 19 skipping in most of our MDS patients (84.1%). By RT-PCR method, we verified exon 4 skipping in *PVRL2* gene and exon 19 skipping in *GRIK5* gene in the 4 MDS patients analyzed (Fig. 5e, f).

## Discussion

Alternative splicing is crucial for normal hematopoiesis; disruption of this physiological process may be related to leukemogenesis^24^. Global aberrant splicing has been reported in many diseases including benign and malignant disorders, such as cystic fibrosis, Alzheimer disease and many cancers, as well as AML^2,5,6,25,26^. Some splicing variants of specific genes have been proposed to serve as diagnostic or prognostic biomarkers for cancer patients^27,28^. Although aberrant AS of specific genes has been reported in MDS, the global pattern of splicing in this disease has not been well explored^29–32^.

Previous studies used expressed sequence tags (ESTs) to identify aberrant AS events^33^, but this method was limited by small numbers of ESTs. Exon array is another tool but lacks comprehensive coverage at the exon-exon junctions^25^. Recently, next generation sequencing-based RNA-seq has been widely used for investigation of alternative splicing, but deep reads are necessary for this purpose^1,34^. One recent study compares Illumina HiSeq 2000 with HTA 2.0 for detection of alternative splicing and concludes that modern microarrays outperform sequencing for standard analysis of gene expression in terms of reproducibility and cost^35^.

In this study, we used BMMNCs rather than a more homogenous population such as sored CD34^+^ cells. A concern is the possible heterogeneity of gene expression profiles and splicing patterns in BMMNCs^36,37^. Nevertheless, in a recent report regarding molecular classification based on gene expression and its correlation with survival and risk of leukemic transformation in MDS, Shiozawa et al. found that the prognostic value of the classification according to the gene expression profiles of BMMNCs was similar to that of the purified CD34^+^ progenitor cells^38^. Further studies are warranted to clarify this concern.

To the best of our knowledge, this is the first report regarding the clinical implications of global splicing aberrancies in MDS patients. By a new platform of arrays containing extensive exon and exon-exon junctional probes, we were able to investigate the genome-wide splicing patterns. We found drastic differences in genome-wide splicing patterns between MDS patients and healthy donors.

Our data revealed 26.9% of expressed genes with aberrant splicing in MDS patients compared to normal BM donors. In general, there were 42,760 aberrant AS events in whole MDS patients (ranged from 24,829 to 87,286 events of each MDS patient) noted at the transcriptome by our HTA 2.0 platform. By contrast, some previous studies with RNA-seq platform reported about 1000 to 125000 aberrant AS events in about 290000 splice junctions examined^39,40^. The reason that the number of aberrant AS events differs among different studies might be explained by different patient populations and study platforms. In addition, these MDS-specific splicing aberrancies were located in both the coding and non-coding regions, as reported by others^5^. It is likely that the aberrant splicing in MDS would lead to alteration of expression in many genes, affect many important cellular processes, and contribute at least partially to the development of this disease.

We quantified the aberrant splicing by “aberrant AS score” defined as total aberrant AS events divided by total aberrant AS genes in each MDS patient. A higher aberrant AS score could reflect a higher degree of genome-wide aberrancies of AS pattern as shown in Fig. 1. With this parameter, we noted the extent of aberrant splicing could predict OS and TTLC in MDS patients, independent of other well established prognostic factors such as age, IPSS-R and important genetic mutations, although it was not different among the risk groups of patients nor was it affected by genetic mutation categories. In a previous study regarding aberrant splicing in AML, CD13 splice variants were reduced in remission but dramatically increased at relapse^5^. However, the prognostic implication of aberrant AS was not studied in that report. In our study, we found a higher degree of aberrancies of AS predicted a poor outcome. Interestingly, a high global aberrant AS score was only significantly associated with *U2AF1* mutation^41^. It is likely that many factors other than gene mutations involving spliceosome or epigenetic modifiers are responsible for the aberrant splicing.

While the degree of AS aberrancies harbored prognostic significance, we were interested in finding specific aberrant AS genes with prognostic significance. These genes may have implications for the pathogenesis of MDS. To this end, we performed a LASSO-Cox regression model to analyze the power of OS prediction among all aberrant AS events and identified 13 events with prominent prognostic significance. The scoring system composed of these 13 specific aberrant AS events could predict both OS and TTLC in MDS patients. These aberrant splicing events might lead to protein loss (nonsense-mediated mRNA decay rule), protein truncation, in-frame peptide insertion/deletion. In addition, alterations in untranslated regions or non-coding RNA are likely to perturb normal gene functions. Among the genes affected, four (*C1QTNF4*, *EGFL7*, *MAP3K15*, *PTK7*) may affect cell proliferation signaling pathway^42–45^; four (*KRT18*, *PVRL2*, *PXDN*, *TGFBI*) can alter cell adhesion leading to inhibition of apoptosis and increasing tumor invasiveness^46–49^; two genes (*ARHGEF17*, *GNAI1*) are supposed to regulate apoptotic pathway^50,51^; HOXA9 is a transcription factor and *MEG3* is a tumor suppressor gene^52,53^. The last gene *GRIK5* forms functional heteromeric kainate-preferring ionic channels while its biological implication for carcinogenesis is unknown yet^54^. Further studies are needed to verify the biological significances of these genes in the pathogenesis of MDS.

In summary, for the first time, our data clearly demonstrated that the complexity of global aberrant splicing had prognostic impacts on MDS patients. Studies in large prospective cohorts are needed to confirm our observations.

## Electronic supplementary material

Supplementary Information
