# Supplementary material for: The prognostic significance of global aberrant alternative splicing in patients with myelodysplastic syndrome
Source: Blood Cancer J. 2018 Aug 13;8(8):78. doi: 10.1038/s41408-018-0115-2 (PMC6089879; doi:10.1038/s41408-018-0115-2)

**Supplementary Table S1**

**Univariate analysis on the overall survival and time to leukemic change**

|  | **Overall Survival** | **Time to leukemic change** |
| --- | --- | --- |
| **Variables** | ***P*** | ***P*** |
| ***Age***^†^ | **0.005*** | **0.369** |
| ***IPSS-R***^§^ | **< 0.001*** | **< 0.001*** |
| *SF3B1* | 0.284 | 0.558 |
| *U2AF1* | 0.865 | 0.428 |
| ***SRSF2*** | **0.032*** | **0.132** |
| ***ZRSR2*** | **0.002*** | **0.353** |
| *TET2* | 0.346 | 0.370 |
| ***ASXL1*** | **0.007*** | **< 0.001*** |
| *DNMT3A* | 0.359 | 0.200 |
| ***EZH2*** | **0.086** | **0.337** |
| *IDH1/IDH2* | 0.528 | 0.006* |
| *RUNX1* | 0.154 | 0.083 |
| ***TP53*** | **< 0.001*** | **< 0.001*** |
| **Aberrant AS score***^Ψ^* | **0.010*** | **0.004*** |

Abbreviation: IPSS-R, revised international prognostic scoring system.

NOTE: variables in bold letters were selected for multivariate analysis.

*Statistically significant (*P* < 0.05)

^†^Age as a continuous variable

^§^IPSS-R risk score > 4.5 relative to IPSS-R ≤ 4.5 (the reference)

*^Ψ^*High global aberrant AS score relative to low global aberrant AS score (the reference)

**Supplementary Table S2**

**Cox regression analysis for the overall survival in 176 MDS patients**

| Factors |  | Hazard ratio | 95% confidence interval | *P* value |
| --- | --- | --- | --- | --- |
| **Mayo Alliance model** | Int-2/High | 2.56 | 1.48-4.40 | 0.001 |
|  | Low/Int-1 | 1.0 (reference) |  |  |
| **Aberrant AS score** | High | 1.78 | 1.03-3.09 | 0.039 |
|  | Low | 1.0 (reference) |  |  |

**Supplementary Table S3**

**Pathway analysis of aberrant AS genes by DAVID database (List of top twenty signaling pathways)**

| **Term-pathways** | **Count** | ***P*-value** | **FDR** | **Fold enrichment** |
| --- | --- | --- | --- | --- |
| **Porphyrin biosynthesis** | 8 | 1.14E-07 | 1.65E-04 | 13.05 |
| **Protein refolding** | 11 | 3.75E-08 | 7.08E-05 | 8.64 |
| **Heme biosynthesis** | 8 | 3.23E-05 | 0.047 | 7.46 |
| **Hemoglobin's Chaperone** | 10 | 5.74E-06 | 0.008 | 5.66 |
| **Chromosome segregation** | 19 | 9.65E-06 | 0.018 | 3.29 |
| **DNA replication** | 22 | 5.61E-06 | 0.008 | 3.09 |
| **Mitosis** | 51 | 8.65E-10 | 1.26E-06 | 2.58 |
| **Lysosome** | 44 | 6.86E-07 | 9.95E-04 | 2.26 |
| **Cell division** | 66 | 9.43E-10 | 1.37E-06 | 2.25 |
| **Cell cycle** | 100 | 1.91E-11 | 2.77E-08 | 2.02 |
| **Immunity** | 70 | 3.43E-08 | 4.98E-05 | 2.01 |
| **Cell-cell adherens junction** | 51 | 9.44E-06 | 0.014 | 1.92 |
| **Cytoskeleton** | 57 | 1.08E-05 | 0.016 | 1.84 |
| **Focal adhesion** | 59 | 9.89E-06 | 0.015 | 1.82 |
| **Protein kinase binding** | 58 | 1.91E-05 | 0.032 | 1.79 |
| **Ubl conjugation** | 229 | 1.27E-15 | 1.78E-12 | 1.68 |
| **ATP-binding** | 178 | 5.79E-12 | 8.40E-09 | 1.67 |
| **Acetylation** | 423 | 1.72E-26 | 2.50E-23 | 1.61 |
| **Protein binding** | 681 | 3.63E-18 | 5.99E-15 | 1.28 |
| **Splice variant** | 752 | 1.53E-16 | 2.11E-13 | 1.26 |

Abbreviation: FDR, false discovery rate.

**Supplementary Table S4**

**Thirteen representative aberrant AS events. The weights were determined by the LASSO-Cox analysis**

| **Transcript ID** | **Gene symbol** | **PSR ID** | **Lasso weight** | **Correlation *P* value** | **Prediction of protein changes** |
| --- | --- | --- | --- | --- | --- |
| TC11000774.hg.1 | *ARHGEF17* | PSR11009971.hg.1 | -0.12 | 3.38E-41 | protein loss or protein truncation |
| TC11001744.hg.1 | *C1QTNF4* | PSR11020433.hg.1 | -0.09 | 1.95E-58 | influence in UTR region |
| TC09000817.hg.1 | *EGFL7* | PSR09010582.hg.1 | -0.13 | 9.79E-89 | influence in UTR region |
| TC07000508.hg.1 | *GNAI1* | PSR07007182.hg.1 | -0.06 | 2.32E-48 | influence in UTR region |
| TC19001564.hg.1 | *GRIK5* | PSR19021309.hg.1 | -0.04 | 2.35E-78 | in-frame protein alteration |
| TC07003401.hg.1 | *HOXA9* | PSR07019143.hg.1 | -0.22 | 7.10E-109 | protein loss or protein truncation |
| TC12000424.hg.1 | *KRT18* | PSR12005401.hg.1 | -0.10 | 1.82E-66 | influence in UTR region |
| TC0X000902.hg.1 | *MAP3K15* | PSR0X012120.hg.1 | -0.03 | 5.73E-87 | protein loss or protein truncation |
| TC14000645.hg,1 | *MEG3* | PSR14007925.hg.1 | -0.08 | 1.51E-78 | influence in lncRNA |
| TC06000595.hg.1 | *PTK7* | PSR06006893.hg.1 | -0.39 | 7.47E-20 | protein loss or protein truncation |
| TC19002164.hg.1 | *PVRL2* | PSR19027172.hg.1 | 0.22 | 2.37E-41 | influence in non-coding RNA |
| TC02001512.hg.1 | *PXDN* | PSR02024632.hg.1 | 0.09 | 1.29E-10 | protein loss or protein truncation |
| TC05000682.hg.1 | *TGFBI* | PSR05009473.hg.1 | 0.09 | 1.49E-25 | protein loss or protein truncation |

Abbreviation: PSR, probe selection region; UTR, untranslated region; lncRNA, Long non-coding RNAs.

NOTE: correlation P value means the P value of each aberrant AS event associated with overall survival.

**Supplementary Figure S1**

**HTA 2.0 analysis workflow in our study**


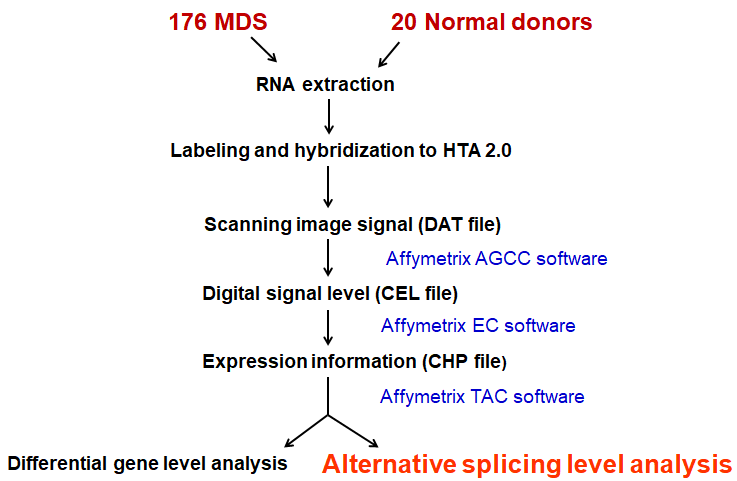


**Supplementary Figure S2**

**Analysis workflow on LASSO-Cox regression model**


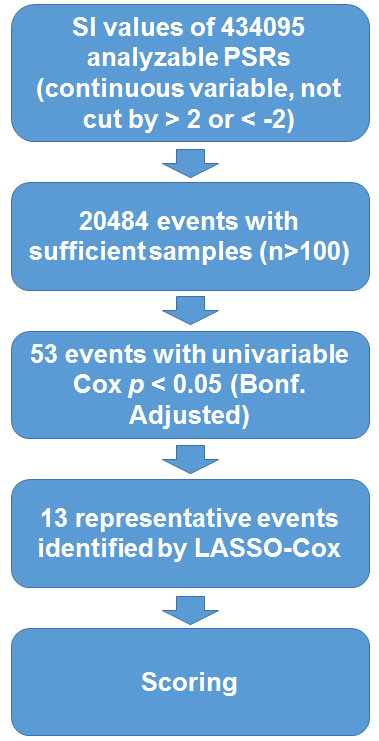


Abbreviation: SI, splicing index; PSR, probe selection region

**Supplementary Figure S3**

**Aberrant AS scores among patients with different splicing factor gene mutations**

**
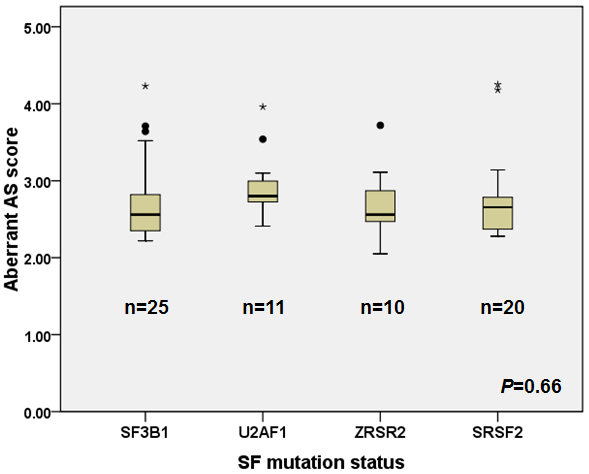
**

Note:

Only the patients with mutation in single splicing factor gene were analyzed and those with mutations in more than one splicing factor genes were excluded. For example, SF3B1 refers to only *SF3B1* splicing factor gene mutation without other spliceosome gene alteration, etc.

．Dots: mild outlier ; * Stars: extreme outlier; bold horizontal lines: median

**Supplementary Figure S4**

**Aberrant AS in *AUP1* would lead to truncation of the original protein in the MDS patient by the prediction of Geneious software**


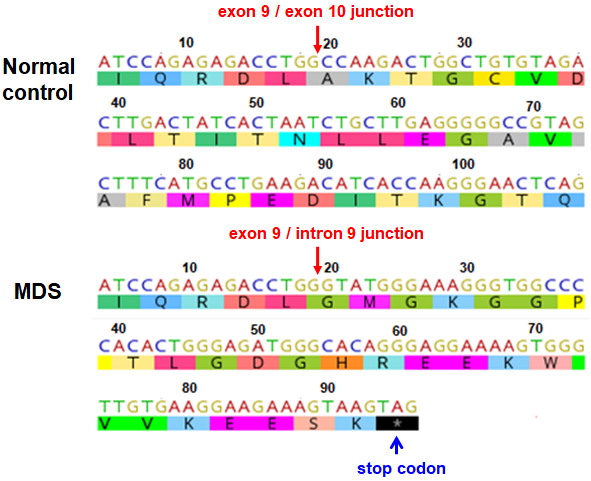

Supplement: Supplementary file 1 — Supplementary Information [file 41408_2018_115_MOESM1_ESM.docx]
